# A PTH Value at 6 Hours Postsurgery Predicts the Diagnosis of Transient and Permanent Hypoparathyroidism

**DOI:** 10.1210/clinem/dgaf416

**Published:** 2025-07-17

**Authors:** Ana Segarra-Balao, Juan de Dios Barranco-Ochoa, María de Damas-Medina, Beatriz Andrea Sánchez-Arquelladas, Eva Antonaya-Rubia, Carmen Rosa-Garrido, María Josefa Martínez-Ramírez, Alberto José Moreno-Carazo

**Affiliations:** Department of Endocrinology and Nutrition, University Hospital of Jaén, Jaén 23007, Spain; Department of Endocrinology and Nutrition, University Hospital of Jaén, Jaén 23007, Spain; Department of Endocrinology and Nutrition, University Hospital of Jaén, Jaén 23007, Spain; Department of Anesthesiology and Resuscitation, University Hospital of Jaén, Jaén 23007, Spain; Department of General Surgery, University Hospital of Jaén, Jaén 23007, Spain; Foundation for Biomedical Research of Eastern Andalusia, Jaén 23007, Spain; Department of Endocrinology and Nutrition, University Hospital of Jaén, Jaén 23007, Spain; Department of Endocrinology and Nutrition, University Hospital of Jaén, Jaén 23007, Spain

**Keywords:** PTH, calcium, hypoparathyroidism, transient, permanent, thyroidectomy

## Abstract

**Context:**

PTH+ levels after thyroid surgery are generally used to detect patients at risk of developing postoperative hypoparathyroidism. However, there is still a lack of consensus about the threshold value regarding its evaluation, the definition of gland function recovery, and the classification of hypoparathyroidism as permanent.

**Objective:**

PTH levels (determined 6 hours after total thyroidectomy) could be effective for early prediction of the risk of postsurgical hypocalcemia and IV calcium requirements during hospitalization, comparing it with the predictive capacity of serum calcium levels at 24 and 48 hours after surgery. We also aim to study the efficacy of the measurement of PTH levels for the predictive diagnosis of permanent hypoparthyroidism.

**Design:**

Prospective cohort study between September 2021 and November 2023.

**Setting:**

A public tertiary care hospital (Jaén, Spain).

**Patients:**

We collected data on 105 patients undergoing total thyroidectomy.

**Main Outcome Measures:**

PTH levels were measured 6 hours postoperatively. Additionally, corrected calcium levels, adjusted for total protein, were measured at 24 hours and 48 hours postsurgery.

**Results:**

In our study, a PTH value at 6 hours postsurgery <10.10 pg/mL suggests, with high sensitivity and specificity, to be a very effective measure for identifying patients who would develop either transient [area under the curve (AUC) = 0.991, 95% confidence interval (CI) 0.978-1] and permanent hypoparathyroidism (AUC = 0.961, 95% CI 0.952-0.997).

**Conclusion:**

Measuring PTH levels at 6 hours postthyroidectomy is an accurate method for predicting which patients are at risk of developing transient and/or permanent hypoparathyroidism.

Hypocalcemia due to hypoparathyroidism is currently considered the most common complication of total thyroidectomy (1). The incidence varies depending on the definition of hypoparathyroidism, with an estimated prevalence of 30% in the immediate postsurgical period (2).

The etiologies include parathyroid gland injury, disruption of blood supply, or inadvertent parathyroid tissue resection. Postsurgical hypoparathyroidism is categorized as either transient or permanent, with the 12-month postoperative period being the most frequently used time marker to distinguish between these forms (3).

This condition holds significant clinical importance. Hypocalcemia can be associated with adverse health-related outcomes and increased healthcare costs, including IV treatment requirements, prolonged hospital stays, and a negative impact on patients’ quality of life (4). Permanent hypoparathyroidism requires lifelong calcium and vitamin D supplementation as well as continuous biochemical monitoring.

Postsurgical hypocalcemia has a delayed onset, typically emerging within 48 to 72 hours following surgery. Thus, serial serum calcium measurements and/or monitoring of hypocalcemic signs and symptoms for at least 72 hours postoperatively are essential for its detection.

To predict its early occurrence, various methods have been developed, including the postsurgery determination of PTH (1, 4, 5). However, neither the optimal timing of assessment nor the definitive PTH cutoff values, whether based on an isolated reading or as a percentage drop from presurgical values, have been clearly established (6-9). Currently, there is limited evidence regarding the utility of postoperative PTH as a predictor of long-term permanent hypoparathyroidism.

Therefore, the primary objective of this study is to assess whether the PTH level (determined 6 hours after total thyroidectomy) can accurately predict the risk of postsurgical hypocalcemia and the need for IV calcium treatment during hospitalization more effectively than calcium levels measured at 24 and 48 hours postsurgery.

The secondary objective is to evaluate the predictive capability of this early PTH measurement for diagnosing a well-defined 1-year mark of permanent hypoparathyroidism.

## Material and Methods

This was a prospective cohort study of consecutive patients undergoing total thyroidectomy at the Hospital Universitario de Jaén (Spain) between September 2021 and November 2023. Patients were followed from the immediate postsurgical period up until 1 year after surgery.

Eligibility criteria included patients over 16 years of age undergoing first-time total thyroidectomy for multinodular goiter, Graves’ disease, or thyroid cancer, as well as those undergoing completion thyroidectomy following a previous hemithyroidectomy. Exclusion criteria included concomitant parathyroidectomy, a history of previous parathyroidectomy, preexisting parathyroid disorders, or subtotal thyroidectomy. The study was approved by our local ethics committee (CEI number: A01037777), in accordance with all applicable regulations. Informed consent was obtained from all participants.

We collected demographic variables such as age and sex, along with clinical variables traditionally associated with the risk of postthyroidectomy hypocalcemia, including the type of thyroidectomy, surgical indication, and extent of lymph node dissection. Postoperatively, serum PTH levels were measured at 6 hours (PTH6h) during the postanesthesia care unit stay. Following transfer to the hospital ward, daily clinical and laboratory monitoring was performed by the endocrinology department, including serum calcium corrected for total protein levels at 24 hours (Ca24h) and 48 hours (Ca48h) postsurgery to ensure normocalcemia.

Calcium supplementation requirements (oral or IV), both during hospitalization and at discharge, were documented. At 1-year follow-up, all patients underwent a comprehensive thyroid evaluation and laboratory testing, including serum PTH and calcium levels.

Calcium levels were determined by the colorimetric 5-nitro-5′-methyl-BAPTA method and intact PTH by electrochemiluminescent immunoassay. The manufacturer of the assays is Roche Diagnostics. PTH (6 hours postoperatively) and calcium levels (24-48 hours postoperatively) were measured in the same laboratory as appropriate. The reference values for calcemia were 8.6 to 10 mg/dL with a coefficient of variation of 3.05% and a lower limit of detection of 0.8 mg/dL; and for intact PTH they were 15 to 65.7 pg/mL, with a coefficient of variation of 3.05% and a lower limit of detection of 2.4 pg/mL.

A postsurgical hypoparathyroidism diagnosis was established based on corrected serum calcium levels under 8 mg/dL and if calcium treatment (oral or IV) was needed during hospital admission and at the time of discharge. Patients with hypocalcemia and elevated PTH were excluded from the study. IV calcium supplementation was deemed necessary if the patient exhibited severe tetany symptoms or if serum calcium levels were under 7.5 mg/dL without associated symptoms, following the protocol outlined in the consensus published by the Spanish Endocrine Society (5). Permanent hypoparathyroidism was defined as protein-corrected calcium below 8 mg/dL or PTH levels below 15 pg/mL while receiving calcium and calcitriol supplementation at the 12-month postsurgical evaluation. The standard treatment protocol at discharge consisted of calcitriol 0.25 to 0.5 mcg daily and oral calcium carbonate 1.25 g (1-6 tablets daily). Calcium citrate was considered as an alternative in cases of absorption difficulties or when high calcium carbonate requirements (>6 g/day) were needed.

Statistical analysis was performed using SPSS version 21. For the descriptive study, we utilized frequencies for qualitative variables and mean and SD for quantitative variables. Appropriate parametric or nonparametric tests (*t*-test, Mann-Whitney U test, chi-square test with Fisher's correction when applicable) were employed when comparing means or frequencies of groups. Additionally, group comparisons were evaluated by binary logistic regression analysis. A *P*-value <.05 was considered statistically significant.

To determine the combined effect of the variables on the diagnosis of transient or permanent hypoparathyroidism, a multivariate logistic regression model was developed using the forward method.

To select the predictors, binary logistic regressions were calculated to evaluate each of the independent variables. From the results of these individual regressions, those variables with a *P*-value of less than .05 were selected as candidate independent variables to be included in the multiple logistic regression study. It is usual to select variables with *P*-values less than .20 (10) but the criteria of Peduzzi (11) recommend using between 10 and 15 events per variable included in the model. For this reason, the number of independent variables was reduced by changing the criterion of *P* less than .20 to *P* less than .05.

To evaluate the quality of the obtained multivariate model, its prognostic capacity for the diagnosis of permanent hypoparathyroidism was assessed using the receiver operating characteristic curve and calculated area under the curve (AUC). Contingency tables were used to determine optimal cutoff values of PTH6h for maximum accuracy, sensitivity, and specificity in predicting both transient and permanent hypoparathyroidism.

## Results

**Figure 1.**
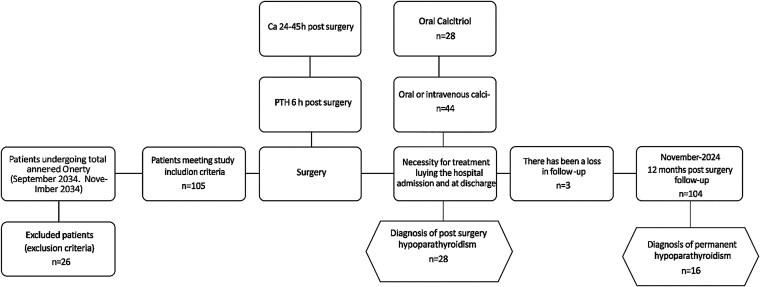
Flow diagram.

We collected data on 105 patients undergoing total thyroidectomy from September 2021. A total of 104 patients who completed the 12-month postsurgical follow-up protocol were evaluated for permanent hypoparathyroidism; we lost 1 patient's data due to death. Figure 1 shows these data in a flow chart. The study population had a mean age of 54.9 ± 14.9 years, comprising 84 females (80%) and 21 males (20%). Surgical indications included suspicion of carcinoma in 53 patients (50.5%), benign goiter in 46 patients (43.8%), and Graves’ disease in 6 patients (5.7%). Total thyroidectomy was performed in 91.4% (96 patients), while only 9 patients (8.6%) underwent completion thyroidectomy following previous hemithyroidectomy. Central neck dissection was performed in 27 patients and lateral neck dissection in 6 patients. Additional postsurgical complications were documented in 8 patients (7.6%). Baseline characteristics for all study subjects are presented in Table 1.

**Table 1.** Baseline characteristics for all subjects in the study

	Total (105)	Female (84)	Male (21)	*P*-value
Age (years), mean ± SD	54.9 ± 14.9	53.4 ± 14.4	61.0 ± 15.9	**.035**
Cause of total thyroidectomy, n (%)				
Tumor	53 (50.5)	42 (50.0)	11 (52.4)	.675
Goiter	46 (43.8)	36 (42.9)	10 (47.6)	
Graves’ disease	6 (5.7)	6 (7.1)	0 (0.0)	
Type of surgery, n (%)				
Hemithyroidectomy to complete a previous one	9 (8.6)	9 (10.7)	0 (0.0)	.199
Total thyroidectomy	96 (91.4)	75 (89.3)	21 (100.0)	
Central neck dissection, n (%)				
No	78 (74.3)	63 (75.0)	15 (71.4)	.955
Yes	27 (25.7)	21 (25.0)	6 (28.6)	
Lateral neck dissection, n (%)				
No	99 (94.3)	79 (94.0)	20 (95.2)	1.000
Yes	6 (5.7)	5 (6.0)	1 (4.8)	
Parathyroid visualization, n (%)				
No	17 (16.2)	13 (15.5)	4 (19.1)	.737
Yes	87 (82.9)	71 (845)	16 (76.2)	
Unknown	1 (0.9)	0 (0.0)	1 (4.7)	
PTH 6 hours, mean ± SD	31.7 ± 33.6	33.4 ± 35.9	25.0 ± 22.1	.358
PTH 6 hours at discharge, n (%)				
<10.10	28 (26.7)	22 (26.2)	6 (28.6)	1.000
=>10.10	74 (70.5)	59 (70.2)	15 (71.4)	
Unknown	3 (2.8)	3 (3.6)	0 (0.0)	
Postoperative complications, n (%)				
No	96 (91.5)	75 (89.3)	21 (100.0)	.354
Yes	8 (7.6)	8 (9.5)	0 (0.0)	
Unknown	1 (0.9)	1 (1.2)	0 (0.0)	
Signs and symptoms of hypocalcemia, n (%)				
No	89 (84.8)	70 (83.3)	19 (90.5)	.518
Yes	16 (15.2)	14 (16.7)	2 (9.5)	
Calcium presurgery, mean ± SD	9.4 ± 0.6	9.5 ± 0.7	9.1 ± 0.3	.371
Calcium corrected 24 hours, mean ± SD	8.9 ± 0.7	8.8 ± 0.7	9.0 ± 0.7	.431
Calcium corrected 48 hours, mean ± SD	8.7 ± 0.8	8.7 ± 0.9	8.9 ± 0.7	.303
Calcium corrected at discharge, mean ± SD	9.0 ± 0.7	9.0 ± 0.7	9.0 ± 0.6	.688
Oral calcium requirement, n (%)				
No	74 (70.5)	58 (69.0)	16 (76.2)	.708
Yes	31 (29.5)	26 (31.0)	5 (23.8)	
Calcitriol requirement, n (%)				
No	77 (73.3)	61 (72.6)	16 (76.2)	.956
Yes	28 (26.7)	23 (27.4)	5 (23.8)	
Intravenous calcium requirement, n (%)				
No	92 (87.6)	72 (85.7)	20 (95.2)	.458
Yes	13 (12.4)	12 (14.3)	1 (4.8)	
Diagnosis of hypoparathyroidism at discharge, n (%)				
No	77 (73.3)	61 (72.6)	16 (76.2)	.956
Yes (with treatment)	28 (26.7)	23 (27.4)	5 (23.8)	
Diagnosis permanent hypoparathyroidism (at 1 year), n (%)				
No	64 (61.0)	50 (59.5)	14 (66.7)	.440
Yes (with treatment)	12 (11.4)	11 (13.1)	1 (4.8)	
Follow-up less than 1 year	29 (27.6)	23 (27.4)	6 (28.5)	

Comparisons were carried out using Fisher's or chi-square tests for qualitative variables and for quantitative variables Student's *t*-test or Mann-Whitney U-test.

The mean presurgery serum calcium value was 9.4 ± 0.6 mg/dL (range 2.4). Postoperative calcium values varied according to measurement timing: 8.9 ± 0.7 mg/dL (range 3.8) at 24 hours, 8.7 ± 0.8 mg/dL (range 3.9) at 48 hours, and 9.0 ± 0.7 mg/dL (range 3.5) at discharge. These data are summarized in Table 2.

**Table 2.** Mean and range measurements of PTH and calcium at each preoperative and postoperative time point

	Total (105)
PTH 6 hours, mean ± SD	31.7 ± 33.6
PTH 6 hours, median [P25-P75] (R)	23.1 [9.0-45.1] (235.5)
Presurgical calcium, mean ± SD	9.4 ± 0.6
Calcium corrected 24 hours, mean ± SD	8.9 ± 0.7
Calcium corrected 48 hours, mean ± SD	8.7 ± 0.8
Calcium corrected at discharge, mean ± SD	9.0 ± 0.7
Presurgical calcium, median [P25–P75] (R)	9.2 [9.0-9.6] (2.4)
Calcium corrected 24 hours, median [P25–P75] (R)	9.0 [8.4-9.4] (3.8)
Calcium corrected 48 hours, median [P25–P75] (R)	8.6 [8.1-9.4] (3.9)
Calcium corrected at discharge, median [P25–P75] (R)	9.0 [8.5-9.4] (3.5)

Abbreviations: P, percentile; R, range.

Postoperative hypoparathyroidism was diagnosed in 28 patients (26.7%). Significant differences were observed between patients who developed transient hypoparathyroidism and those who did not, specifically in calcium levels at 24 hours (Ca24h; 8.24 ± 0.57 mg/dL vs 9.09 ± 0.58 mg/dL; *P* < .001), calcium levels at 48 hours (Ca48h; 8.09 ± 0.90 mg/dL vs 9.03 ± 0.58 mg/dL; *P* < .001), PTH6h (5.64 ± 2.7 pg/mL vs 41.03 ± 34.63 pg/mL; *P* < .001), and the rate of central neck dissection (44.4% vs 20.5%; *P* = .023).

Permanent hypoparathyroidism was diagnosed in 16 patients (15.4%) among the 104 patients who completed annual follow-up. Similarly, when comparing patients who developed permanent hypoparathyroidism vs those who did not, statistically significant differences were found in Ca24h (8.32 ± 0.51 vs 8.97 ± 0.67; *P* < .001), Ca48h (8.26 ± 1.07 vs 8.84 ± 0.72, *P* = .015), PTH6h (4.92 ± 2.41 vs 36.67± 34.29; *P* < .001), and central neck dissection (25.9% vs 11.7%; *P* = .118).

To determine the diagnostic accuracy of postoperative levels of PTH6h and central neck dissection in predicting transient and permanent hypoparathyroidism, the multivariate model identified PTH6h as the sole significant predictor for both transient and permanent hypoparathyroidism (Tables 3 and 4).

**Table 3.** Results of regression study for transient hypoparathyroidism

Univariate logistic regression									
Variable	n	B	SE	Wald	Degrees of freedom	*P*-value	Odds ratio	95% confidence interval for odds ratio	
								Lower limit	Upper limit
Sex (male) Reference category: female	105	−0.188	0.568	0.109	1	.741	0.829	0.272	2.522
Age (years)	105	−0.020	0.015	1.877	1	.171	0.980	0.952	1.009
Cause of total thyroidectomy	105			2.080	2	.353			
Tumor	53	−0.057	0.915	0.004	1	.950	0.944	0.157	5.672
Goiter	46	−0.721	0.942	0.585	1	.445	0.486	0.077	3.085
Graves’ disease	6	Reference category							
Central neck dissection	105	1.131	0.478	5.599	1	.018	3.100	1.214	7.913
Lateral neck dissection	105	0.339	0.896	0.143	1	.705	1.404	0.243	8.122
PTH 6 hours (pg/mL)	102	−0.886	0.309	8.227	1	.004	0.412	0.225	0.755
Multivariate logistic regression									
PTH 6 hours (pg/mL)	98	−0.885	0.309	8.183	1	.004	0.413	0.225	0.757

Abbreviations: B, regression coefficient; n, sample size.

**Table 4.** Results of regression study for permanent hypoparathyroidism

Univariate logistic regression									
Variable	n	B	SE	Wald	Degrees of freedom	*P*-value	Odds ratio	95% confidence interval for odds ratio	
								Lower limit	Upper limit
Sex (male) Reference category: female	76	−1.125	1.087	1.070	1	.301	0.325	0.039	2.735
Age (years)	76	−0.030	0.020	2.123	1	.145	0.971	0.933	1.010
Cause of total thyroidectomy	76			0.888	2	.641			
Tumor	39	Due to the small sample size, statistics cannot be calculated.							
Goiter	33								
Graves’ disease	4	Reference category							
Central neck dissection	76	1.520	0.656	5.376	1	.020	4.573	1.265	16.533
Lateral neck dissection	76	1.036	1.268	0.668	1	.414	2.818	0.235	33.809
PTH 6 hours (pg/mL)	73	−0.477	0.169	7.941	1	.005	0.620	0.445	0.865
Multivariate logistic regression									
PTH 6 hours (pg/mL)	70	−0.477	0.170	7.933	1	.005	0.620	0.445	0.865

Higher PTH6h levels demonstrated a protective effect against developing permanent hypoparathyroidism. For each unit increase in PTH6h, the risk of diagnosis decreases by 38.0% [odds ratio 0.62, 95% confidence interval (CI) 0.495-0.830], with this risk reduction being statistically significant (*P* = .001).

**Figure 2.**
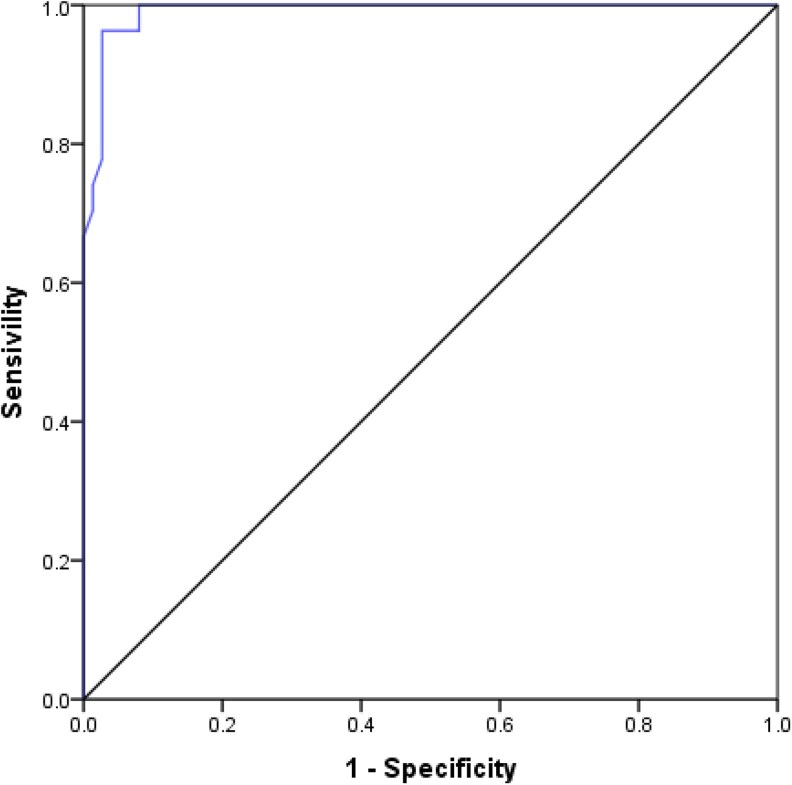
Receiver operating characteristic curve of PTH 6 hours for transient hypoparathyroidism.

For transient hypoparathyroidism, the model correctly classified 99.1% of patients (AUC = 0.991, 95% CI 0.978-1.000). The optimal cutoff value for PTH6h that best discriminated between patients with and without transient hypoparathyroidism was determined to be 10.10 pg/mL. This threshold identified 99% of patients who developed hypocalcemia requiring IV calcium supplementation during hospitalization (Fig. 2).

**Figure 3.**
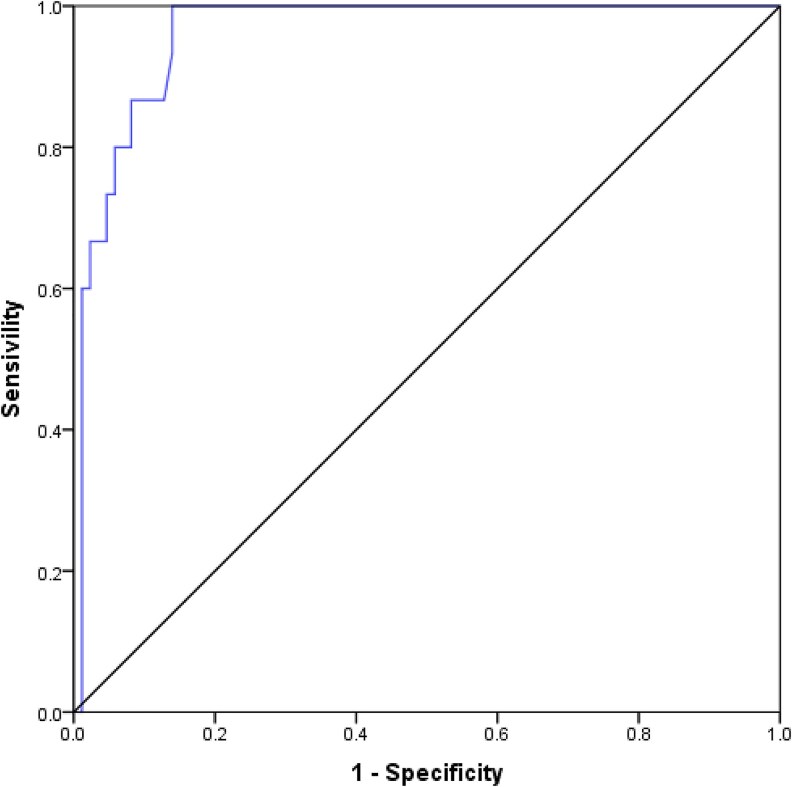
Receiver operating characteristic of PTH 6 hours for permanent hypoparathyroidism.

For permanent hypoparathyroidism, PTH6h demonstrated excellent discriminatory ability with an AUC of 0.961 (95% CI 0.925-0.997). The optimal PTH6h cutoff point for predicting permanent hypoparathyroidism was also determined to be 10.10 pg/mL. All patients with PTH6h values below this threshold were diagnosed with permanent hypoparathyroidism within 1 year of follow-up (Fig. 3).

## Discussion

Hypoparathyroidism (transient and permanent) represents a highly prevalent complication that poses a significant clinical challenge for endocrinology units monitoring patients undergoing total thyroidectomy. Accurately predicting which patients may develop transient and/or permanent complications is crucial for optimizing patient management, reducing hospital stay duration, and minimizing healthcare costs associated with this procedure (9, 12-14).

Despite numerous research efforts addressing this issue, the optimal approach for identifying patients at risk of developing postsurgical hypoparathyroidism remains undefined.

Multiple studies have explored factors associated with complications following thyroid surgery, including patient characteristics (age, sex), gland pathology (enlargement, fibrosis, inflammation), surgical variables (extent of thyroidectomy, lymph node dissection) (15), procedural factors (surgical extensiveness, repeated interventions), and surgeon expertise (15, 16). Various predictors of immediate postthyroidectomy hypocalcemia have been identified, including single postoperative intact PTH values, percentage decrease of intact PTH from baseline, 25-hydroxyvitamin D levels, and underlying thyroid disorders such as Graves’ disease (17-19).

Some clinicians advocate prophylactic calcium supplementation for all postthyroidectomy patients, resulting in unnecessary treatment for approximately 70% of patients.

Postoperative PTH levels serve as excellent predictors of hypocalcemia. However, until recently, the percentage decrease in PTH levels after surgery has been the most commonly studied parameter, necessitating logistically challenging serial analytical determinations within short timeframes in the context of scheduled surgeries. The optimal timing for postsurgical PTH determination has not been clearly established when utilizing a single measurement approach (20-22).

Previous studies have examined PTH measurements at various intervals (0 hours, 4 hours, 12 hours, 24 hours) primarily for diagnosing rather than predicting hypoparathyroidism. Furthermore, standardized PTH cutoff points have not been established, and improved follow-up strategies for hypoparathyroidism classification as permanent, based on well-defined diagnostic criteria, are needed (7).

Monitoring PTH levels during patient follow-up should ideally predict parathyroid function recovery and guide decisions regarding discontinuation of replacement therapy. This requires a more precise diagnosis of permanent hypoparathyroidism (7, 18) and more accurate postsurgical PTH cutoff points than those determined in previous retrospective studies (5).

This study demonstrates that measuring a single PTH value at 6 hours postthyroidectomy—without requiring preoperative PTH baselines or calculating absolute/relative percentage decreases—provides superior predictive value for accurately defined hypoparathyroidism compared to corrected calcium values determined during the first 48 hours postsurgery. Additionally, this approach effectively identifies patients likely to require IV calcium supplementation during hospitalization. Early identification and treatment of hypocalcemia facilitate reduced hospital stays and appropriate discharge planning. To our knowledge, this represents the first prospective evaluation of PTH at 6 hours postthyroidectomy as a predictor of permanent parathyroid gland damage.

Our study has limitations, including a small sample size and challenges in standardizing PTH cutoff values as a single-center investigation. Center-specific cutoff values and establishing a precise timing for postoperative PTH determination that is applicable to clinical practice would likely be necessary for broader implementation. In conclusion, our findings introduce an easily reproducible preventive protocol based on a single PTH determination in the immediate postsurgical period, enabling more effective monitoring of patients at high risk for hypocalcemia while potentially reducing hospitalization duration and associated healthcare costs.

## Data Availability

Data supporting this study are included in the article and/or supporting materials.

## Abbreviations

AUC: area under the curve
Ca24h: corrected calcium by total proteins at 24 hours
Ca48h: corrected calcium by total proteins at 48 hours
PTH6h: PTH at 6 hours postintervention
